# Effectiveness of laxatives in elderly - a cross sectional study in nursing homes

**DOI:** 10.1186/1471-2318-11-76

**Published:** 2011-11-17

**Authors:** Gunvor S Fosnes, Stian Lydersen, Per G Farup

**Affiliations:** 1 Department of Medicine, Innlandet Hospital Trust, Gjøvik, Norway; 2 Unit for Applied Clinical Research, Department of Cancer Research and Molecular Medicine, Norwegian University of Science and Technology, Trondheim, Norway; 3 Department of Research, Innlandet Hospital Trust, Gjøvik, Norway

## Abstract

**Background:**

Laxatives are efficient drugs, but the effectiveness has been questioned. In nursing homes, the prevalence of constipation is high and laxatives are commonly used drugs. The aims of the study were to assess the effectiveness of laxative therapy in an everyday setting in Norwegian nursing homes, study differences between treatment regimens and factors associated with normal bowel function.

**Methods:**

A cross-sectional study. After giving informed consent, residents above 60 years of age using laxatives for functional constipation were included, and their characteristics, medical history, use of drugs and bowel functions were recorded. Normal bowel function was defined as bowel movements from 3 times/week to 3 times/day and stool consistency 3-5 on Bristol Stool Form Scale.

**Results:**

Out of 647 residents in the nursing homes, 197 were included and 116 (59%) had normal bowel function. The treatment effect did not differ significantly between the laxatives, treatment regimens or expected efficacy of the regimens. The treatment was unsatisfactorily adapted to individual needs. In subjects with normal bowel function, 113 (97%) had persistent complaints; 68 (59.5%), 10 (8.0%), 34 (28.6%) and 26 (22.5%) reported straining, manual manoeuvre to facilitate bowel movements, feeling of incomplete bowel movements, and feeling of anorectal obstruction respectively. Good nutritional status, previous or present cancer disease and anxiety/depression were predictors of normal bowel function.

**Conclusions:**

Treatment of constipation in nursing homes was unsatisfactory. Nearly all patients with normal stool frequency and consistence had some persistent complaints. Improved nutrition and individualization of the treatment could improve the outcome.

## Background

The prevalence of constipation in nursing homes is up to 74% and more than half of the residents use laxatives [1,2]. Constipation is most often a primary or functional disorder of unknown aetiology associated with gut dysmotility (slow transit) and pelvic floor dysfunction, but might be secondary to an organic disease. Symptoms are infrequent defecations, hard stools, straining, sensation of incomplete defecation, sensation of anorectal obstructions, and need for manual manoeuvres to facilitate bowel movements [3]. After exclusion of organic diseases, these symptoms are the basis for the diagnosis of functional constipation according to the Rome criteria [4]. Constipation is a troublesome disorder associated with reduced quality of life and high costs [2,5,6].

Laxatives are, in addition to conservative interventions (dietary fibre, physical activity, fluid etc.), the cornerstone in the treatment of constipation. All groups of laxatives (osmotic laxatives, stimulant laxatives, enemas/suppositories, and miscellaneous pharmaceuticals) are superior to placebo [7]. But in contrast to the overall good results in clinical trials, patients' satisfaction with everyday use of laxatives is low, only 47% were satisfied in a web-based survey in the general population [8]. Knowledge of the treatment of constipation in frail elderly is insufficient and the treatment poses extra challenges in this population [3,9].

The aims of this cross sectional study in nursing homes were to asses the effectiveness of laxative therapy in an everyday setting, study differences between treatment regimens and search for factors associated with satisfactory effect.

## Methods

### Study design and methods

In 2008-2009, this cross sectional study was performed in nursing homes in the counties of Oppland and Hedmark, Norway.

Registered and auxiliary nurses completed case report forms based on information from the participants and their next of kin and in the medical records. A blood sample was collected.

### Study population

Residents above 60 years of age using laxatives regularly or on demand and who had stayed in the nursing home for more than 8 weeks, were eligible for the study. Patients with known organic gastrointestinal diseases (malignancy, stenosis/stricture, inflammatory bowel disease, intestinal resection etc.) that could be related to constipation and those with a planned discharge within two weeks were excluded.

### Variables

#### General characteristics

The following variables were recorded: age, gender, weight, height, smoking habits, use of alcohol, somatic and psychiatric diseases, mobility (score 0-2: Bedridden or sitting in a chair, walk indoors, walk outdoors), Katz' Activity of Daily Living (ADL) (index 0-6: 0 = Very dependent; 6 = Independent), nutritional status (Mini Nutritional Assessment (MNA^® ^) score 0-30: <17 malnourished, 17-23.5 at risk of malnutrition, 24-30 normal nutritional status) [10], diet (fibre, number of glasses of fluid/day, consistency of food (mashed food/soups, bread without crust, ordinary food)), all use of drugs (registered according to the Anatomical Therapeutic Chemical Classification System (ATC) level 5) [11], number of drugs, and use of one or more drugs with markedly anticholinergic effect (defined as group 3 according to Carnahan et al) [12]. A blood sample was analysed for haematological and biochemical screening (electrolytes, hepatic and renal diseases, and thyroid function).

#### Use of laxatives

The use and dosage of laxatives was recorded at ATC-level 5. Groups of laxatives were defined at ATC-level 4: osmotically acting laxatives (A06AD); contact laxatives (A06AB); bulk laxatives (A06AC); enemas (A06AG); and softeners/emollients (A06AA). The dosage of each laxative was graded as on demand, regular use standard dose, and regular use of high dose. High dose was defined as: liquid paraffin > 15ml/day; bisacodyl > 10mg/day; senna glycosides > 24mg/day; sodium pico sulphate: > 10 drops (5 mg)/day; lactulose > 30ml/day; macrogol combinations: > 26.2 (2 sachets); docusate sodium > 1 supp/day; and laurilsulfate: > 1supp/day. An overall grading of the laxative effect of the regimens from low to high was as follows: on demand treatment only; regular use of only fibre or lactulose; regular use of only contact laxatives, enemas, polyethylene glycol or liquid paraffin; and use of at least two laxatives of which one was used regularly. Proper use of laxatives was assessed by comparison with generally accepted treatment recommendations [2,13-15].

#### Bowel function

Defecation frequency (number of stools per day), stool consistency (Bristol Stool Form Scale score 1-7) [16], straining, sensation of incomplete evacuation, sensation of anorectal obstruction/blockade, and manual manoeuvres to facilitate bowel movements, were recorded. Normal bowel function was defined as defecation frequency from three defecations/week to three defecations/day and stool consistency 3-5 on Bristol Stool Form Scale.

### Statistical analyses

Comparisons between groups were performed with student t-test, Mann-Whitney U test, unconditional z-pooled test for table analyses with small counts (≤ 5) [17], exact chi-square and linear-by-linear dependent on type of variable and normality. Manual backward logistic regression analyses with a one-by-one stepwise removal of the least significant variable were performed with "bowel function" (normal/altered) as dependent variable. Independent variables were variables with at least 10 subjects in the smallest group and associated with bowel function with p ≤ 0.2 in the bivariate analyses. Since a drug is associated with the disease under treatment, both the group of drugs and the disease were included if one of them was associated with bowel function in the bivariate analyses. The one with the lowest impact on the outcome in the multivariable analyses was removed. Age, gender and variables with p < 0.05 were maintained in the equation.

Multiple imputations for missing data were performed with a model including all principal variables [18]. The statistical analyses were performed with PASW statistics 18 and StatXact. Two-sided p-values ≤ 0.05 were regarded as statistically significant.

### Ethics

All participants or their next of kin gave informed consent to participate. The study was approved by the Regional Committee for Medical and Health Research Ethics, Central Norway and The Norwegian Data Inspectorate, represented by Privacy Ombudsman for Research at Oslo University Hospital, Ullevål, and performed in accordance with the Declaration of Helsinki.

## Results

### Participants

**Figure 1:**
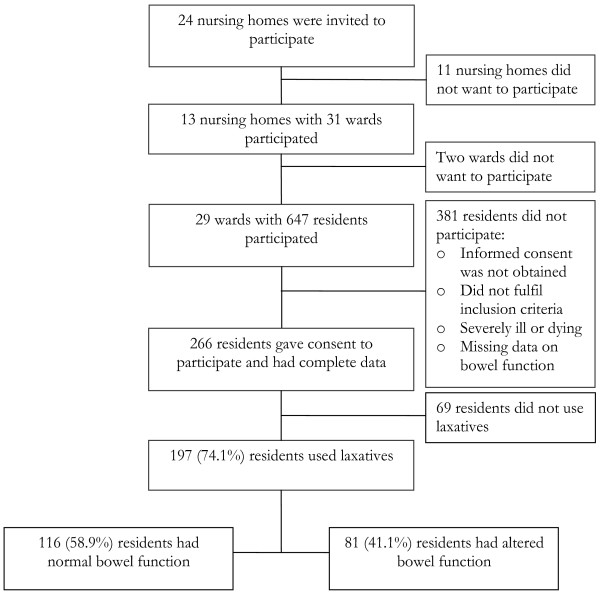
**The participants in the study**.

The study included 197 residents using laxatives regularly or on demand. Figure 1 is a flow chart of the residents. The mean age of the participants was 85.6 years (sd 6.9; range 67-103 years), 147 (74.6%) were women, 123 (62.4%) were unable to give informed consent, and 109 (53.3%) were bedridden. One hundred and sixteen (58.9%) had normal bowel function. Table 1 gives the characteristics of the participants in more detail. Three residents with gastro-intestinal cancer were excluded.

**Table 1:** Characteristics of the participants and a comparison between participants with and without normal bowel function.

Characteristics	All participants n = 197	Participants with normal bowel function n = 116	Participants with altered bowel function n = 81	Statistics p-value
Age (years)	85.6 (SD 6.9)	85.1 (SD 7.1)	86.4 (SD 6.7)	0.18
Gender - female	74.6	71.6	79.0	0.25
Body mass index (kg/m^2^) (n = 183)	24.3 (SD 5.3)	24.9 (SD 5.3)	23.5 (SD 5.2)	0.07
Smoking: Current/Before/Never (n = 193) (%)	7.8%/16.1%/76.2%	10.4%/17.4%/72.2%	3.8%/14.1%/82.1%	0.07
Use of alcohol more than once a month (n = 193)	29 (15.0%)	14 (12.3%)	15 (19.0%)	0.22
Intake of liquids (glass/day)	9.0 (1.0-17.0)	9.0 (3.0-15.0)	8.0 (1.0-17.0)	0.01
Dietary fibre (g/day) (n = 195)	14.5 (3.7-41.0)	14.7 (4.6-41.0)	13.5 (3.7-30.3)	0.15
Katz: Activity of daily living^1^	1 (0-6)	1 (0-6)	1 (0-6)	0.31
Mobility: Bedridden/Walk indoors/Walk outdoors	105(53.3%)/54(27.4%)/ 38(19.3%)	61(52.6%)/28(24.1%) /27(23.3%)	44(54.3%)/26(32.1%)/11(13.6%)	0.36
MNA^2 ^- score (n = 141)	20.0 (8.5-26.0)	20.8 (8.5-26.0)	18.0 (9.5-26.0)	0.05
Consistency of food (n = 196) Mashed food/Bread without crust/Ordinary food	35(17.9%)/39(19.9%) /122(62.2%)	15(12.9%)/20(17.2%) /81(69.8%)	20(25.0%)/19(23.8%) /41(51.3%)	0.007
Number of diseases (n = 196)	5.0 (1-15)	5.0 (1-15)	4.0 (0-11)	0.36
Heart diseases (n = 195)	86 (44.1%)	52 (45.2%)	34 (42.5%)	0.77
Thrombo-embolic disease (n = 195)	16 (8.2%)	6 (5.2%)	10 (12.5%)	0.11
Stroke (n = 195)	75 (38.5%)	44 (37.9%)	31 (39.2%)	0.88
Depression/anxiety (n = 196)	98 (50.0%)	65 (56.0%)	33 (41.3%)	0.06
Dementia (n = 196)	104 (53.1%)	58 (50.0%)	46 (57.5%)	0.31
Diabetes (both I and II) (n = 196)	22 (11.2%)	13 (11.2%)	9 (11.3%)	1.00
Parkinson's disease(n = 196)	11 (5.6%)	5 (4.3%)	3 (7.5%)	0.34
Hypothyroidism (n = 195)	8 (4.1%)	5 (4.3%)	3 (3.8%)	0.84
Rheumatoid arthritis (n = 196)	8 (4.1%)	2 (1.7%)	6 (7.5%)	0.04
Cancer (n = 191)	30 (15.7%)	24 (21.4%)	6 (7.6%)	0.01
Abdominal operation (n = 193)	30 (15.5%)	22 (19.6%)	8 (9.9%)	0.07
Number of drugs	6 (0-20)	6 (0-20)	5(0-17)	0.10
Antithrombotic agents (B01A)	104 (52.8%)	61 (52.6%)	43 (53.1%)	1.00
Iron preparations (B03A)	35 (17.8%)	23 (19.8%)	12 (14.8%)	0.45
Diuretics (C03)	88 (44.7%)	54 (46.6%)	34 (42.0%)	0.56
Calcium channel blockers (C08)	23 (11.7%)	13 (11.2%)	10 (12.3%)	0.83
Thyroid hormones (H03AA)	16 (8.1%)	10 (8.6%)	6 (7.4%)	0.80
Antibiotics (J01)	28 (14.2%)	19 (16.4%)	9 (11.1%)	0.41
NSAID (M01A)	14 (7.1%)	9 (7.8%)	5 (6.2%)	0.67
Opioids (N02A)	24 (12.2%)	14 (12.1%)	10 (12.3%)	1.00
Dopaminergic agents (N04B)	9 (4.6%)	2 (1.7%)	7 (8.6%)	0.02
Benzodiazepin derivatives (N05BA)	46 (21.8%)	32 (27.6%)	14 (13.6%)	0.02
Antidepressants (N06A)	85 (43.1%)	50 (43.1%)	35 (43.2%)	1.00
Drug with anticholinergic effect	15 (7.6%)	8 (6.9%)	7 (8.6%)	0.79
High dose of ≥1 laxatives	71 (40.1%)	41 (40.6%)	30 (39.5%)	1.00

^1 ^Katz - Activity of daily living. (Index 0-6: 0 = Very dependent; 6 = Independent)

^2 ^MNA = Mini nutritional assessment^® ^(score 0-30: <17 malnourished, 17-23.5 at risk of malnutrition, 24-30 normal nutritional status)

n = number available for the analysis

The results are given as mean (SD), median (range) and number (%). There were no missing values for the use of drugs.

### Use of laxatives

Fourteen (7.1%) used laxatives on demand only, 162 (82.2%) used laxatives regularly only and 21 (10.7%) used laxatives on demand in addition to regular use. Table 2 gives the dosage schedule of the various laxatives. The most frequently used laxatives were lactulose, sodium pico sulfate and bisacodyl, used by 124 (63.5%), 41 (21.3%) and 24 (12.2%) respectively. Twenty participants had incomplete data on the dosage schedule. The dosage schedules of bisacodyl, senna glycosides, sodium pico sulphate, macrogols and enemas were usually one to three times a week.

**Table 2:** Dosage schedules of the laxatives.

Substance	ATC-level 5	Dosage schedules			Missing data
			
		On demand	Regular use standard dose	Regular use high dose	
Liquid paraffin	A06AA01	1 (11.1%)	5 (55.6%)	2 (22.2%)	1 (11.1%)
Bisacodyl	A06AB02	2 (7.7%)	21 (80.8%)	1 (3.8%)	2 (7.7%)
Senna glycosides	A06AB06	3 (17.6%)	9 (52.9%)	0	5 (29.4%)
Sodium pico sulphate	A06AB08	5 (10.6%)	36 (76.6%)	3 (6.4%)	3 (6.4%)
Ispaghula (psylla seeds)	A06AC01	1 (14.3%)	-	-	6 (85.7%)
Lactulose	A06AD11	14 (10.1%)	100 (72.5%)	10 (7.2%)	14 (10.1%)
Macrogol combinations	A06AD65	1 (5.9%)	16 (94.1%)	0	0
Docusate sodium	A06AG10	0	5 (100.0%)	0	0
Laurilsulfate	A06AG11	11 (52.4%)	10 (47.6%)	0	0

The results are given as number of participants with proportion (%) in brackets.

Twenty participants had incomplete data on the dosage of regularly used laxatives, and several participants used more than one drug.

In subjects without normal bowel function, generally accepted treatment guidelines were disregarded in two out of 6 (33%) with hard and lumpy stools who did not use osmotic laxatives and in 3 out of 3 subjects (100%) with defecation less than once a week who did not use prokinetics or contact laxatives. In 27 subjects in need of manual manoeuvres to facilitate a bowel movement, 24 (89%) did not use enemas and 9 (33%) did not use osmotically acting laxatives.

### Effect of laxatives

Normalization of the bowel function, which was achieved by 116 (58.9%), did not differ significantly between the laxative regimes or the potency of the regimens (tables 3 and 4). In subjects with normal bowel function, 113 (97%) had persistent complaints; straining, manual manoeuvres to facilitate bowel movements, feeling of incomplete bowel movements and feeling of anorectal obstruction were reported by 68 (59.5%), 10 (8.0%), 34 (28.6%) and 26 (22.5%) respectively.

**Table 3:** Effect of regular use of groups of laxatives

Laxatives	Normal bowel function n = 108 (59.0%)	Altered bowel function n = 75 (41.0%)
Osmotically acting laxatives only	59 (64.8%)	32 (35.2%)
Contact laxatives only	16 (55.2%)	13 (44.8%)
Bulk only	2 (100.0%)	0 (0%)
Enemas only	2 (66.7%)	1 (33.3%)
Softeners/emollients only	2 (33.3%)	4 (66.7%)
Combination of laxatives	27 (51.9%)	25 (48.1%)

The results are given as number of participants with proportion (%) in brackets.

There were no significant differences between the effect of the groups of laxatives (exact chi-square test p = 0.36)

**Table 4:** Bowel function related to expected efficacy of the laxative regimen.

	Normal bowel function n = 116 (59%)	Altered bowel function n = 81 (41%)
On demand only	8 (57.1%)	6 (42.9%)
Lactulose or fibre only	52 (66.7%)	26 (33.3%)
One laxative other than lactulose and fibre	29 (54.7%)	24 (45.3%)
Two or more laxatives	27 (51.9%)	25 (48.1%)

The results are given as number of participants with proportion (%) in brackets.

There were no significant differences between the groups (exact chi-square p = 0.34; and linear-by-linear p = 0.16)

### Predictors for normal bowel function

Table 1 gives the characteristics of participants with and without normal bowel function and comparisons between the groups. Low intake of fluids, poor nutritional status, intake of mashed food and use of dopaminergic agents were statistically significantly associated with altered bowel function; and cancer (previous or present), rheumatoid arthritis and use of benzodiazepines with normal bowel function. Good nutritional status, cancer and depression/anxiety were independent predictors of normal bowel function in the multivariable analyses (table 5). Neither abnormal biological parameters nor the use of drugs with marked anticholinergic effect was associated with the bowel function.

**Table 5:** Independent predictors for altered bowel function.

	Statistics		
	
	OR^1^	CI^2^	p-value
Gender (female)	1.46	0.69 - 3.07	0.32
Age (years)	1.01	0.96 - 1.06	0.67
Cancer disease (previous or present)	0.26	0.09 - 0.71	< 0.01
MNA^3 ^sum score	0.89	0.81 - 0.97	< 0.01
Anxiety/depression	0.46	0.24 - 0.86	0.02

Stepwise backward logistic regression analyses

^1 ^Odds ratio

^2 ^95% confidence interval

^3 ^Mini Nutritional Assessment^® ^(score 0-30: <17 malnourished, 17-23.5 at risk of malnutrition, 24-30 normal nutritional status)

## Discussion

### Effect of laxatives

In this study, 81/197 (41%) of residents in nursing homes treated for constipation did not achieve normalization of stool frequency and consistency. This was judged as unsatisfactory, but was at least as good as in clinical trials reporting 40-85% non-responders [7]. Comparisons are, however, difficult because the definitions of satisfactory response vary [7]. The effect did not differ significantly between the drugs and regimens, and was unrelated to the assumed potency of the regimens, which supports the finding from a systematic review that there is no evidence for the superiority of one laxative to another [19]. Better understanding of the etiology and pathophysiology of constipation could allow individual adjustment of the treatment and improve the success rates [3].

In accordance with others, we found that nearly all subjects with normalized stool frequency and consistence had persistent and bothersome symptoms such as straining, feeling of incomplete bowel movements and anorectal obstruction, and some were in need of manual manoeuvres to facilitate defecation [3]. In all, this pragmatic study showed that ordinary treatment of constipation in elderly is far from desirable.

### Choice of laxatives

All laxatives used in this study have been proven to be superior to placebo [7]. Lactulose was the most frequently used laxative and the only one regularly used for long-term treatment in high doses. The frequent use of lactulose, despite the rather high price, depends on local therapeutic tradition. The drug was for a long time the only osmotic laxative on the market in Norway, and was believed to have fewer side effects during long-term treatment. The definitions of high doses for continuous treatment has, however, not been defined and was arbitrarily chosen [13,14,20-22]. Some new treatment alternatives (such as lubiprostone and prucalopride) were not available, and could perhaps have improved the outcome for some participants.

Although all regimens are known to be effective, and that one is not superior to another, tailoring the regimens to the individual subject could probably improve the outcome. Guidelines propose algorithms for treatment of constipation [7]. Some guidelines differ between acute and chronic constipation, degrees of constipation, and different subgroups such as slow transit, normal transit, anorectal outlet obstruction, constipation in pregnancy etc.[2,13-15]. In this study, these guidelines were not in regular use and the treatment was neither adapted to the cause of constipation nor sufficiently individualized. All patients were probably given the same initial treatment, and depending on the effect, the dose was increased or a new regimen added depending on local traditions. This explains why a significant proportion of subjects with hard stools did not use osmotically acting laxatives, why subjects with infrequent bowel movements did not use prokinetics (which was not available) or contact laxatives, and why subjects in need of manual manoeuvres to facilitate a bowel movement did not use enemas or osmotically acting laxatives, which are recommended treatments for these complaints [2,13-15].

The prescription of laxatives is the physicians' responsibility, but in daily practice registered nurses often accomplish this treatment rather independently. Increased involvement of physicians and tailoring of the treatment to the individual patient could probably improve the outcome. The insufficient understanding of the actual patho-physiology in the individual subject makes tailoring of the treatment difficult.

### Independent predictors for altered bowel function

Numerous factors have been associated with constipation [1,15,23,24]. In this study, as reported by others, reduced nutritional status was an independent predictor of altered bowel function [1,25]. Efforts to achieve optimal nutrition in elderly are of importance for their general health and could relieve constipation. Previous or present cancer and anxiety/depression were associated with normal bowel function. Except for an association between mental distress and diarrhoea, these findings are difficult to interpret and of limited clinical importance [26,27]. Anticholinergics have been associated with constipation, but in this study, drugs with marked anticholinergic effects were not associated with the effectiveness of laxatives.

### Strengths and limitations

This pragmatic study showed the effectiveness of everyday treatment of constipation in an unselected nursing home population with inclusion of residents regardless of cognitive functions and co-morbidities. This study describes real life, which we think is a strength and a valuable supplement to the optimal results shown in well conducted clinical trials with conscious follow up, perfect adjustment of the treatment regimens and exclusion of many patients. The results in this study correspond well with those in clinical trials [7].

The inclusion of nearly everyone, including frail and mentally reduced participants, necessarily reduced the data quality. Information about symptoms were obtained from the participants themselves (some degree of cognitive reduction was common), their next of kin (some had limited knowledge about their relatives) and the nurses (with variable knowledge of the participant and different clinical judgement). Since bowel functions are in focus for residents at these ages in nursing homes and the investigators informed about the study and the necessity of correct data, the data quality has been judged as satisfactory. The participation rate was low but the characteristics of the participants seem to be representative for residents in nursing homes.

The use of laxatives might have been imprecisely registered. Some nursing homes might have had an uncontrolled use of lactulose, and laxatives in general were handled more roughly and less accurately than other drugs and could have been given without registration.

The pathophysiology of constipation is complex and the laxatives' way of action is incompletely understood. Therefore, factors contributing to constipation and the effect of laxatives might have been missed or under-estimated.

## Conclusion

Treatment of constipation in nursing homes was unsatisfactory and independent of treatment regimen. Out of 197 residents, only 116 (59%) achieved normalization of stool frequency and consistency, and even in this group, 113 (97%) had persistent bothersome symptoms. Focus on individualized therapy based on pathophysiology and specific symptoms, more involvement of physicians and better follow-up, together with use of new laxatives, could improve the outcome of this common and bothersome disorder.

## Competing interests

The authors declare that they have no competing interests.

## Authors' contributions

GSF and PGF designed the study. GSF has been responsible for collection of the data, has worked up the data file, performed the statistical analyses under supervision of the medical statistician (SL), interpreted the results, and written the manuscript. SL is responsible for the medical statistics and has participated in interpretation of the results and preparation of the manuscript. PGF is the main responsible and supervisor for the project and has participated in all parts of the study. All authors have read and approved the last version of the manuscript.

## Pre-publication history

The pre-publication history for this paper can be accessed here:

http://www.biomedcentral.com/1471-2318/11/76/prepub
